# Acute epiglottitis as the initial presentation of pediatric Systemic Lupus Erythematosus

**DOI:** 10.1186/1546-0096-7-19

**Published:** 2009-10-31

**Authors:** Sirirat Charuvanij, Kristin M Houghton

**Affiliations:** 1 Division of Pediatric Rheumatology, British Columbia's Children's Hospital, Vancouver, Canada; 2 University of British Columbia, Vancouver, Canada

## Abstract

We report a case of a 5-year old girl, who initially presented with acute epiglottitis, sepsis and multi-organ failure. She was subsequently diagnosed as having Systemic Lupus Erythematosus. To the best of our knowledge, this article describes the first case of *Haemophilus influenzae *type f epiglottitis as the initial presentation of SLE in childhood.

## Introduction

Systemic Lupus Erythematosus (SLE) is a periodic, multisystem, autoimmune disease characterized by the presence of antinuclear antibodies. Approximately 15% of SLE cases are diagnosed before the age of 16 and children often have more severe disease at onset and higher rates of organ involvement than adults [1]. We report a case of SLE with initial presentation of *Haemophilus influenzae *epiglottitis. Laryngeal involvement and pneumococcal epiglottitis have infrequently been reported in the adult literature [2-4]. This is the first pediatric case and the first case of *Haemophilus influenzae *epiglottitis reported in SLE.

## Case presentation

**Figure 1:**
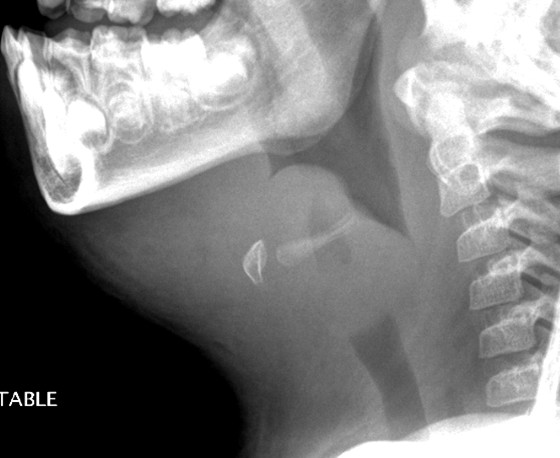
**A lateral neck radiograph demonstrates a profoundly swollen epiglottis and complete airway obstruction**.

A 5-year old fully immunized and previously healthy girl of Filipino origin presented to her local hospital with a one day history of high fever (39°C), sore throat, stridor and shortness of breath. She was transferred to BC Children's Hospital and was intubated by an otolaryngologist in the operating room. Subsequently, she was admitted to the ICU and was intubated and ventilated for 3 days. A lateral neck radiograph revealed a profoundly swollen epiglottis and complete airway obstruction (Figure 1). The diagnosis of acute epiglottitis was made based on her clinical picture and imaging findings. She was empirically treated with intravenous Cefotaxime. Her throat swab and blood culture were positive for *Haemophilus influenzae *type f.

On day 2 of admission, she developed multi-organ system dysfunction with anemia, thrombocytopenia, acute renal failure and hypertension (BP 123/80 mmHg). Investigations revealed hemoglobin at 63 g/L, white blood cell 7 × 10^9^/L with lymphocyte of 1.85 × 10^9^/L. Her platelets were 53,000/mm^3^. DAT was positive. PT and INR were normal. APTT and dilute Russell Viper time were elevated at 41 sec (20.2-32.9) and 2.17 ratio (0.76-1.15) respectively. Fibrinogen and D Dimer were elevated. Her serum creatinine was 143 umol/L (<51). Urinalysis showed 10-20 RBC/hpf, protein 0.3 g/L with hyaline and granular casts. Treatment included supportive care, antibiotic (Cefotaxime), red blood cell and platelet transfusions.

**Figure 2:**
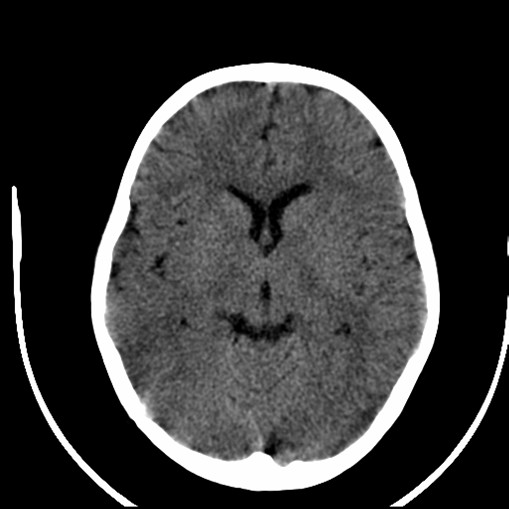
**CT brain reveals multiple areas of abnormal hypodensity in the subcortical white matter of both hemispheres**.

**Figure 3:**
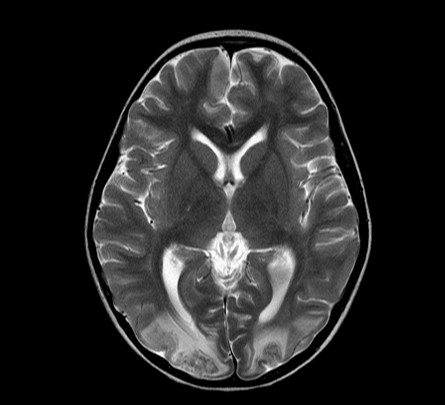
**MRI brain shows extensive T2 signal changes throughout the white matter with diffuse cerebral edema involving both cerebral hemispheres**.

On day 7 of admission, she developed seizures which were felt to be due to severe hypertension (BP 170/100 mmHg). Computed tomography (CT) brain revealed multiple areas of abnormal hypodensity in the subcortical white matter of both hemispheres (Figure 2). Magnetic resonance imaging (MRI) brain demonstrated extensive T2 signal changes throughout the white matter with diffuse cerebral edema involving both cerebral hemispheres (Figure 3). A diagnosis of probable posterior reversible encephalopathy syndrome (PRES) was made.

On day 9 of admission, the rheumatology team was consulted. The main differential diagnosis at that time included SLE, Thrombotic Thrombocytopenic Purpura (TTP) and Wegener's Granulomatosis. A week later, her autoimmune and serologic workup came back positive for anti-nuclear antibody (ANA) at a titer of >1: 1280, positive anti-double stranded DNA, lupus anticoagulant, anticardiolipin antibody (ACA IgG was elevated at 6.1 MoM) and low complements C3 (0.14 g/L) and C4 (0.04 g/L). She underwent a renal biopsy which showed mesengial lupus nephritis (WHO class II) but due to a poor sample (only 4 glomeruli on light microscopy) the possibility of active proliferative lupus nephritis could not be excluded. Based on her clinical and laboratory findings, she was diagnosed with SLE and treated with a course of pulse Methylprednisolone 30 mg/kg/day for 3 days and subsequently converted to high dose oral prednisone (2 mg/kg/day). She received her first Cyclophosphamide infusion (500 mg/m^2^/dose) in the hospital prior to discharge.

She was discharged home on prednisone (2 mg/kg/day) with a scheduled taper, Hydroxychloroquine, Enalapril, Amlodipine and Clonazepam. She was scheduled to receive Cyclophosphamide infusions as an outpatient. At last assessment in our rheumatology clinic (9 months after initial presentation), her disease was in clinical remission. Her anti dsDNA was negative. C3 was slightly low at 0.71 g/L. C4 was normal. ENA panel remained negative. She continued to have positive ANA at lower titer of 1: 640. She completed 8 courses of monthly Cyclophosphamide infusions and was switched to Mycofenolate Mofetil for maintenance immunosuppressant therapy.

## Discussion

Epiglottitis is an inflammation of the epiglottis and the adjacent tissues surrounding the epiglottis. The clinical presentation includes abrupt onset of high fever, severe sore throat, dysphagia, "hot potato" voice, drooling and rapid progression of airway obstruction. Several bacteria have been associated with epiglottitis in children. Among them, *Haemophilus influenzae *type b was responsible for most cases of epiglottitis in pediatric patients in the pre- Hib vaccine era [5].

*Haemophilus influenzae *is a pleomorphic gram-negative coccobacillus. There are encapsulated and non-encapsulated forms. After the initiation of Hib protein-polysaccharide conjugate vaccines, most cases of invasive *Haemophilus influenzae *infection have been attributed to non-type B strains [6]. Serology type f (Hif) is reported as the most common cause of invasive encapsulated non-b *Haemophilus influenzae *disease in children [7]. The risk of invasive *Haemophilus influenzae *disease include younger and older ages, complement deficiency, hypogammaglobulinemia, sickle cell anemia, functional asplenia, malignancy and HIV infection.

SLE is the prototypical multisystem autoimmune disease. Clinical manifestations of SLE are variable and the most common pediatric presentations include arthritis, malar rash, nephritis and central nervous system disease [8]. Our patient had lupus nephritis, hematological abnormalities, a positive ANA and a positive antibody to double stranded DNA, thus fulfilling four of the revised American College of Rheumatology classification criteria for SLE [9]. TTP was also initially considered given her thrombocytopenia, anemia, coagulopathy, fever, renal involvement and CNS involvement. TTP associated with SLE has been described [10-12]. However, her peripheral smear did not show typical microangiopathic changes of TTP and ADAMTS 13 was not performed. Furthermore, her seizure activity was thought to be due to PRES, not TTP or CNS lupus but the clinical presentations have considerable overlap and definitive diagnosis is challenging. PRES is a rapidly developing neurologic condition characterized by headache, decreased alertness, visual loss, seizures, hypertension and lesions in the posterior cerebral white matter. PRES was first reported in 1996 by Hinchey [13]. PRES associated with SLE has been reported in adults and children [13-17]. Our patient's MRI brain showed multiple hypodensity lesions in the subcortical white matter of both hemispheres, right post-central gyrus and on the left just at the central sulcus in keeping with the effects of hypertensive encephalopathy ("PRES").

Apart from the infection risk associated with immunosuppressive therapies, infection in SLE can be secondary to a primary defect in the immune system or alterations in the innate and adaptive immune responses. In addition, splenic dysfunction is reported in SLE patients [18,19]. One study reported 4.6% of SLE patients with functional asplenia [19]. These patients seem to have increased susceptibility to encapsulated bacteria including pneumococcal, meningococcal, *Haemophilus influenzae *and salmonella infection. Complement abnormalities are also reported in SLE patients resulting in defective opsonization of encapsulated organisms [20]. Even though infections occur commonly in SLE patients, it is rarely the presenting feature of the disease. The presentation of our patient with *Haemophilus influenzae *type f epiglottitis is unusual.

Although our patient did not have morphologic erythrocyte changes, it is possible that she developed splenic dysfunction during her initial clinical presentation of SLE. This may explain an increased susceptibility to *Haemophilus influenzae*. However, a 99m-technetium sulphur colloid scan and 99m- technetium labeled, heat-damage erythrocyte scan were not performed to support this hypothesis. The other hypothesis is that SLE is induced by *Haemophilus influenzae *infection. It is reported that bacterial DNA can enhance several of the autoimmune abnormalities observed in SLE and perhaps play a pathogenic role in the induction of SLE [21]. There is a report of *Haemophilus influenzae *type f pericarditis and tamponade as the initial manifestation of systemic lupus erythematosus in an adult [22]. There are no reports of epiglottitis as the initial presentation of SLE in the literature but there are case reports of pneumococcal epiglottitis in adults with SLE and lupus overlap syndrome [2,3]. Laryngeal involvement in adult SLE ranges from mild ulcerations, vocal cord paralysis, laryngeal edema to necrotizing vasculitis with airway obstruction [4]. Furthermore, severe upper airway obstruction from cricoarythenoiditis was reported as the presenting manifestation of a SLE flare in an adult [23].

In conclusion, our pediatric patient presented with *Haemophilus influenzae *type f epiglottitis and septicemia and was subsequently diagnosed as having SLE. An opsonization defect and splenic dysfunction are most likely responsible for her increased susceptibility to infection with encapsulated bacteria. In addition, it is postulated that severe bacterial infection may play a role in inducing SLE. SLE has been called the "disease with a 1,000 faces" as it can present in many ways and it needs to be considered in the differential diagnosis of a sick child with multi-system disease. Clinicians need to be vigilant for infection in children with SLE as they may be at increased risk from their disease, functional hyposplenism and immunosuppressant therapy. Indeed, infection is the leading cause of death in pediatric SLE [24]. Our clinical practice is to vaccinate all newly diagnosed SLE patients against encapsulated organisms.

## Competing interests

The authors declare that they have no competing interests.

## Authors' contributions

SC and KH participated in drafting the manuscript. All authors read and approved the final manuscript.

## Consent

Consent was obtained from the parent of the patient for publication of this case report and accompanying images.
